# The novel ciprofol versus propofol during gastroenteroscopy in Chinese patients: a meta-analysis

**DOI:** 10.3389/fmed.2025.1598268

**Published:** 2025-06-25

**Authors:** Xiaoyan Liang, Qing Feng, Guozhen Li, Tong Liu, Lingyuan Zeng, Yang Xiao, Kun Zhang

**Affiliations:** ^1^School of Public Health, Xinjiang Medical University, Urumqi, China; ^2^Anesthesia Surgery Center, West China Hospital, Sichuan University, Chengdu, Sichuan, China; ^3^The Second People’s Hospital of Pidu District, Chengdu, China; ^4^Department of Anesthesiology, Jingzhou Hospital Affiliated to Yangtze University, Jingzhou, Hubei, China

**Keywords:** ciprofol, propofol, gastroscopy, colonoscopy, adverse drug events, Chinese participants

## Abstract

**Introduction:**

Gastrointestinal endoscopic interventions have become a routine practice in healthcare institutions. Propofol has been the most preferred general anesthetic agent for gastrointestinal endoscopic interventions. Ciprofol is a novel compound that has been approved by the China Medical Products Administration for sedation during gastrointestinal endoscopic procedures since December 2020. In this analysis, we aimed to systematically compare ciprofol versus propofol during gastroenteroscopy in Chinese patients.

**Methods:**

The search databases included MEDLINE, EMBASE, Google scholar, Web of Science, http://www.Clinicaltrials.gov, and the Cochrane database. Studies which were based on Chinese participants were included in this analysis. The procedural outcomes and adverse drug events were considered as the endpoints. Statistical analysis was carried out by the RevMan software version 5.4. Risk ratios (RR) with 95% confidence intervals (CI) were used to represent results for dichotomous data whereas weighted mean differences (WMD) with 95% CI were used to represent the result for continuous data.

**Results:**

Twelve studies with a total number of 2055 Chinese participants (enrolled from 2017 to 2023) were included in this analysis whereby 1073 participants were assigned to ciprofol and 982 participants were assigned to propofol. Following the administration of anesthetic agent, the results for the induction time [WMD: 0.33; (95% CI: −0.64 to 1.30); *P* = 0.50], awake time [WMD: 0.42, (95% CI: 0.03–0.81); *P* = 0.03], duration of gastroenteroscopy [WMD: 0.22; (95% CI: −0.09 to 0.53); *P* = 0.16] and recovery time [WMD: 0.48; (95% CI: 0.13–0.83); *P* = 0.007] were not significantly worse with ciprofol compared to propofol. In addition, ciprofol was associated with a significantly lower risk of injection pain (RR: 0.15, 95% CI: 0.08–0.27; *P* = 0.00001), respiratory depression (RR: 0.49, 95% CI: 0.36–0.74; *P* = 0.0003), hypotension (RR: 0.76, 95% CI: 0.64–0.90; *P* = 0.001) and drowsiness (RR: 0.75, 95% CI: 0.57–0.98; *P* = 0.04). The risk for nausea and vomiting (RR: 0.95, 95% CI: 0.50–1.80; *P* = 0.87), bradycardia (RR: 1.03, 95% CI: 0.60–1.76; *P* = 0.92) and dizziness (RR: 0.93, 95% CI: 0.63–1.36; *P* = 0.70) were also not increased with ciprofol when compared to propofol in these Chinese patients.

**Conclusion:**

Through this meta-analysis, it could be concluded that ciprofol was apparently not associated with significantly worse procedural outcomes nor associated with increased adverse drug events compared to propofol during gastroenteroscopy in Chinese patients. However, in view of several limitations in this analysis, this hypothesis should further be confirmed in future studies.

## Introduction

Gastrointestinal endoscopic interventions have become a routine practice in healthcare institutions all around the globe (1). Gastrointestinal endoscopies enable early diagnosis and detection of diseases of the gastrointestinal tract and the digestive system with their diagnostic and therapeutic approach which is fast, effective and less invasive (2). However, procedural analgosedation during gastroenteroscopy has become a vital part and is considered the gold standard of gastrointestinal endoscopies.

Even though advances in medicine have reached heights, the type and the amount of sedation which is administered is affected by the nature of the procedure and several patient factors. However, a guideline for the use of anesthetic agents during gastrointestinal endoscopic interventions has been established by the British Society of Gastroenterology Endoscopy Committee (3). Choice of the anesthetic agent is among the several challenges faced by the doctors providing this service. An ideal analgosedation technique should be cost-effective, ensure maximum safety and should ensure satisfaction by the patients.

Till date, propofol has been used as the gold standard anesthetic agent for such procedures, and it has been the most preferred general anesthetic agent for gastrointestinal endoscopic interventions (4). However, propofol is associated with several adverse drug reactions (5). Therefore, new researches are focusing on other newer alternatives to propofol as sedation for such procedures (6).

In this new era, remimazolam and ciprofol are potential newer anesthetic agents (7). Future studies are focusing on these anesthetic agents. Today, these newer potential anesthetic agents are in the focus of researchers’ interest.

Ciprofol is a novel compound that has been developed in China in the year 2017 by the Haisco Pharmaceutical Group Company Limited and it has been approved by the China Medical Products Administration for sedation during gastrointestinal endoscopic procedures since December 2020 (8).

In this analysis, we aimed to systematically compare the outcomes observed with the anesthetic agent ciprofol versus propofol during gastroenteroscopy in Chinese patients.

## Materials and methods

### Search databases and search strategies

Studies were searched from July 2024 till May 2025. The search databases included MEDLINE, EMBASE, Google scholar, Web of Science,^1^ and the Cochrane database.

References of selected publications were also checked for relevant studies.

The following search terms were used:

– Ciprofol versus propofol;
– Ciprofol versus propofol and gastroenteroscopy;
– Ciprofol versus propofol and gastroscopy;
– Ciprofol versus propofol and colonoscopy;
– Ciprofol and propofol and procedures.

### Inclusion and exclusion criteria

Studies were included if:

(a) They were randomized trials comparing ciprofol versus propofol for gastroenteroscopy;
(b) They reported procedural related endpoints or adverse drug events as endpoints;
(c) They were published in English.

Studies were excluded if:

(a) They were systematic reviews, meta-analyses, and reviews of the literature;
(b) They did not report data which compared ciprofol versus propofol for gastroenteroscopy;
(c) They were duplicated studies based on the same trial.

### Data extraction and quality assessment

The authors independently extracted data from the original studies. Authors’ names, participants’ enrollment time period, year of publication, any associated co-morbidities, the endpoints, number of events, the number of participants assigned to each particular group, the type of study, the methodological quality of each study, the baseline features of the studies were all carefully extracted. Any disagreement which occurred were carefully discussed and solved by consensus.

The quality assessment of the trials was carried out by the recommendations suggested by the Cochrane collaboration and this assessment was represented through a diagram (9). The following components were assessed including random sequence generation, allocation concealment, blinding of participants and personnel, blinding of outcome assessment, incomplete outcome data and selective bias.

### Statistical analysis

Statistical analysis was carried out by the RevMan software version 5.4. Heterogeneity was assessed by the (1) Q statistic test; (2) I^2^ statistic test. A subgroup analysis with a *P*-value less or equal to 0.05 was considered statistically significant whereas a subgroup analysis with a *P*-value greater than 0.05 was considered insignificant statistically. For the I^2^ statistic test, a higher value of I^2^ denoted a higher heterogeneity whereas a lower value of I^2^ denoted a lower heterogeneity.

A random effects model was used to represent data during analysis. Random effects models are particularly useful when there is significant heterogeneity (variations) among the groups or the outcomes being studied. This random effects model was used to account for the fact that several factors including different hospital settings, different patient characteristics, variations in treatments which could influence the final treatment outcomes. Therefore, by incorporating random effects, the model could estimate how much of the overall variation in the dependent variable is due to differences between the groups. In addition, random effects models could allow for the generalizability of the findings to a larger population since the model estimates how the effects of the independent variables might vary across different levels rather than just focusing on one aspect. For example, in the case of this study, where we are studying the comparison of ciprofol versus propofol during gastroenteroscopy in Chinese patients, when using random effects model for data from different original studies that were included, the model could estimate how the outcomes might vary across different studies. In addition, this random effects model was also considered since it uses a process called “shrinkage” where estimates for individual groups are “pulled” toward a common average, based on the amount of variation between the groups. This shrinkage can be particularly helpful when you have small sample sizes within some groups, as it allows you to “borrow strength” from other groups with larger sample sizes.

For dichotomous data, the number of events within each outcome was reported, and for continuous data, the mean and standard deviation were provided for analysis. Risk ratios (RR) with 95% confidence intervals (CI) were used to represent data for dichotomous data whereas weighted mean differences (WMD) with 95% CI were used to represent the result for continuous data.

Sensitivity analysis was also carried out to show whether the result of this analysis was influenced by data from any particular original study. This sensitivity analysis was carried out by an exclusion method whereas each original study was excluded one by one and a new analysis was carried out each time to observe for any significant difference in results.

Publication bias was also assessed through funnel plots. This meta-analysis consisted of a smaller number of studies, therefore, by observing the funnel plot, publication bias could be assessed. We have considered the funnel plot to visually assess publication bias since the Egger’s test is not recommended to be used in meta-analyses with a limited number of studies. With a small number of studies, the Egger’s test may not have sufficient power to distinguish between a true publication bias and random variation which meant that it might fail to detect bias when a bias exists (false negative) or it might incorrectly suggest bias when a bias does not exist (false positive). Moreover, in meta-analyses with a limited number of studies and with studies having a lower sample size, the presence of a few small studies might disproportionately influence the test results. While the Egger’s test provides a numerical indicator, with the limited number of studies and studies with smaller population size, Egger’s test’s power to detect asymmetry may be too low to be meaningful. Hence, Egger’s test has not been carried out, rather, visual inspection of the funnel plot was considered crucial for assessing publication bias in this study.

### Ethical approval

This meta-analysis is based on previously conducted studies and does not contain any study with human participants or animals performed by any of the authors. Therefore, an ethical approval was not required for this manuscript.

## Results

### Search outcomes

Similar to other meta-analyses, the preferred reporting items in systematic reviews and meta-analyses (PRISMA) guideline was followed (10). A total number of 78 publications were obtained. After a careful evaluation of the titles and abstracts, 24 publications were eliminated since they were not related to the scope of this research article. A total number of 54 full-text articles were assessed for eligibility. Further eliminations were carried out based on the criteria for inclusion and exclusion for the following reasons:

(a) Systematic reviews and meta-analyses (4);
(b) Based on hysteroscopy and gynecological procedures (3);
(c) Based on general anesthesia (4);
(d) Based in the intensive care unit set ups (6);
(e) Duplicated studies (25).

**FIGURE 1:**
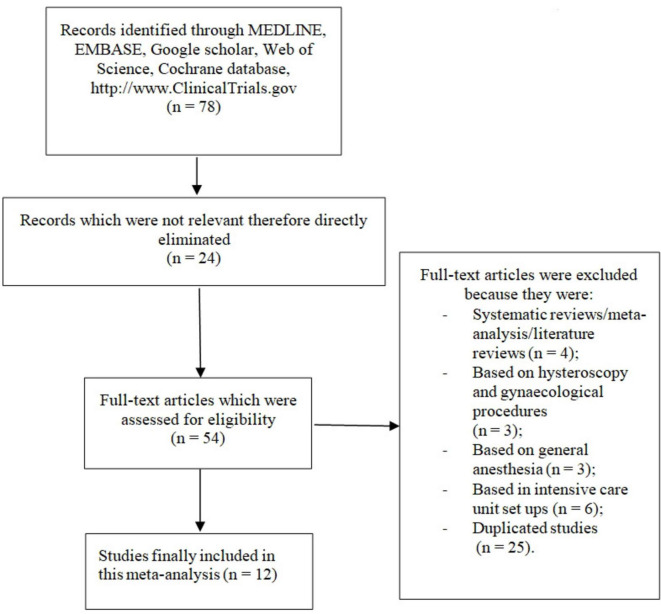
Flow diagram demonstrating the study selection.

Finally, 12 studies (7, 11–21) were selected to be used in this analysis. The flow diagram for the study selection has been illustrated in Figure 1.

### Main features of the included studies

Eight studies with a total number of 2,055 participants (enrolled from 2017 to 2023) were included in this analysis whereby 1,073 participants were assigned to ciprofol and 982 participants were assigned to propofol as shown in Table 1. All the patients were randomly assigned to the respective sedative agent prior to gastroenteroscopy. The participants were of Chinese origin.

**TABLE 1:** The main features of the studies.

Studies	Type of participants’ assignment	Time period of participants’ enrollment (years)	No of participants assigned to ciprofol (n)	No of participants assigned to propofol (n)	Country
Chen et al. (11)	Random	2021–2022	47	49	China
Chen et al. (12)	Random	2022	105	44	China
Gao et al. (13)	Random	2021–2022	82	82	China
Li et al. (14)	Random	−	144	145	China
Li et al. (15)	Random	2023	108	109	China
Liao et al. (16)	Random	2021–2022	185	183	China
Liu et al. (17)	Random	2022–2023	30	30	China
Zhao (18)	Random	−	56	56	China
Teng et al. (19)	Random	2017–2018	63	32	China
Zhang et al. (20)	Random	2021–2022	93	92	China
Zhang et al. (21)	Random	2023	40	40	China
Zhou et al. (7)	Random	2023	120	120	China
Total no of participants (n)			1,073	982	

**FIGURE 2:**
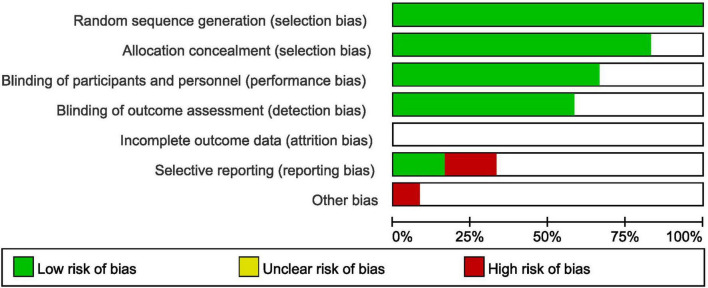
The bias risk assessment based on the recommendations by the Cochrane collaboration.

The methodological quality of the trials has been assessed with reference to the recommendations suggested by the Cochrane Collaboration and this assessment has been illustrated in Figure 2.

### Endpoints assessed in this analysis

The endpoints which were assessed in this analysis included:

(a) Induction time (time taken for the anesthetic drug to cause sedation);
(b) Operation time (duration of the surgery);
(b) Awake time (time taken by the patient to become fully awake post-gastroenteroscopy);
(d) Recovery time;
(e) Injection pain;
(f) Nausea and vomiting;
(g) Respiratory depression;
(h) Hypotension;
(i) Bradycardia;
(j) Dizziness;
(k) Drowsiness;
(l) Total adverse events;
(m) Severe adverse events.

The endpoints which were reported in the original studies have been listed in Table 2.

**TABLE 2:** The endpoints which have been reported in the original studies.

Studies	Endpoints	Type of procedure
Chen et al. (11)	Induction time, operation time, waking time, directional force recovery time, injection pain, total incidence of adverse events	Gastroenteroscopy
Chen et al. (12)	Nausea and vomiting, respiratory depression, injection pain, operation time, time of awakening	Gastroenteroscopy
Gao et al. (13)	Onset time, recovery time, injection pain, respiratory depression, apnea, hypotension, bradycardia, tachycardia, drowsiness, dizziness, nausea and vomiting	Colonoscopy
Li et al. (14)	Induction time, fully alert time, recovery time, total adverse events, mild, moderate and severe adverse events, serious adverse drug reactions, serious adverse events, elevated conjugated bilirubin, prolongation of QT interval, respiratory depression, apnea, hypoxemia, pain on injection, sinus bradycardia, hypotension, dizziness	Gastroenteroscopy
Li et al. (15)	Induction time, recovery time, awake time, choking, injection pain, involuntary movement, nausea and vomiting, dizziness, bradycardia, hypotension, severe hypotension, airway intervention	Gastroscopy
Liao et al. (16)	Motility, choking, hypotension, hypoxemia, bradycardia, dizzy, nausea, vomit, injection pain	Gastroscopy
Liu et al. (17)	Induction dosage, induction time, procedural time, awakening time, injection pain, muscle tremor, body movements	Gastroscopy
Zhao (18)	Nausea and vomiting	Gastroscopy
Teng et al. (19)	Time to fully alert, adverse events, mild, moderate and severe adverse events, drug related adverse events, adverse events associated with sedation, serious adverse event, hypotension, sinus bradycardia, airway obstruction, pain at injection site	Colonoscopy
Zhang et al. (20)	Induction time, procedural time, overall cardiopulmonary adverse events, overall cardiovascular disorders, hypotension, hypertension, overall cardiac disorders, bradycardia, tachycardia, arrhythmia, overall respiratory disorder, respiratory depression, apnea, injection pain, time to successful induction, awakening time, recovery time	Gastroscopy and colonoscopy
Zhang et al. (21)	Respiratory related adverse events, respiratory depression, apnea, hypoxemia, hypotension, hypertension, bradycardia, injection pain, dizziness, nausea/vomiting, movement during procedure	Gastroscopy
Zhou et al. (7)	Dizziness, drowsiness, hypotension, hypoxia	Gastroenteroscopy

### Baseline features of the studies

The baseline features of the participants were represented in Table 3. Based on the data shown in Table 3, the mean age of the participants varied from 38.5 to 54.0 years. The mean percentage of male patients varied from 34.9 to 88.8% as shown in Table 3. The body mass index varied from 22.2 to 30.2 kg/m^2^ and the mean systolic blood pressure varied from 104 to 135 mmHg, and the mean diastolic blood pressure varied from 63 to 87 mmHg as shown in the Table 3.

**TABLE 3:** The baseline features of the participants.

Studies	Age (years)	Males (%)	BMI (kg/m^2^)	DM (%)	Mean BP (mmHg)
	C/P	C/P	C/P	C/P	C/P
Chen et al. (11)	43.2/41.2	34.9/46.8	23.5/25.2	−	[127/87], [135/87]
Chen et al. (12)	48.4/43.6	42.9/40.9	22.7/22.2	−	[132/75], [135/75]
Gao et al. (13)	54.0/54.0	41.5/39.0	23.4/23.7	−	[106/64], [104/64]
Li et al. (14)	43.8/44.1	38.2/43.4	23.2/23.4	−	−
Li et al. (15)	46.4/47.3	50.0/45.0	23.2/23.4	17.6/21.1	[133/76], [133/63]
Liao et al. (16)	45.0/45.4	88.8/72.6	23.1/23.1	−	−
Liu et al. (17)	45.6/45.0	43.3/60.0	23.0/23.7	−	[123/68], [125/68]
Zhao (18)	38.5/38.5	52.7/52.7	22.4/22.4	−	−
Teng et al. (19)	43.5/47.4	41.3/51.6	22.8/23.4	−	−
Zhang et al. (20)	−	−	−	−	−
Zhang et al. (21)	44.5/48.0	65.0/65.0	29.8/30.2	−	−
Zhou et al. (7)	48.0/48.7	43.3/55.0	−	−	−

C, Ciprofol; P, Propofol; BMI, Body mass index; DM, Diabetes mellitus; BP, Blood pressure.

### Main results of this analysis

Results of this analysis were summarized in Table 4.

**TABLE 4:** The main results of this analysis.

Endpoints	RR or WMD with 95% CI	P value	*I*^2^-value (%)
Induction time	0.33 [−0.64 to 1.30]	0.50	98
Awake time	0.42 [0.03 to 0.81]	0.03	89
Operation time	0.22 [−0.09 to 0.53]	0.16	62
Recovery time	0.48 [0.13–0.83]	0.007	82
Injection pain	0.15 [0.08–0.27]	0.00001	68
Respiratory depression	0.49 [0.36–0.67]	0.0003	0
Hypotension	0.75 [0.64–0.90]	0.01	15
Drowsiness	0.75 [0.57–0.98]	0.04	0
Nausea and vomiting	0.95 [0.50–1.80]	0.87	51
Bradycardia	1.03 [0.60–1.76]	0.92	0
Dizziness	0.93 [0.63–1.36]	0.70	64
Total adverse events	0.88 [0.68–1.15]	0.35	81
Serious adverse events	0.42 [0.14–1.24]	0.12	11

RR, Risk ratios; CI, Confidence intervals; WMD, Weighted mean difference.

**FIGURE 3:**
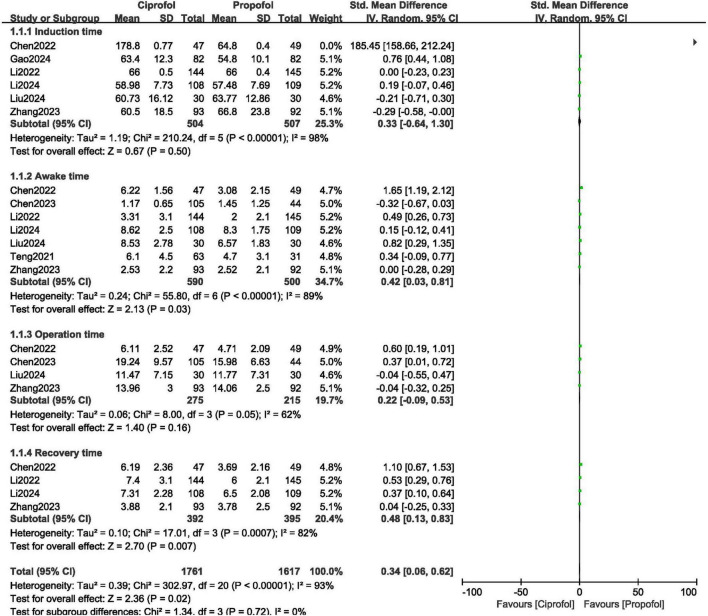
Procedural outcomes observed with ciprofol compared to propofol when sedating patients in gastroenteroscopy.

Following the administration of anesthetic agent, the results for the induction time [WMD: 0.33; (95% CI: −0.64 to 1.30); *P* = 0.50], awake time [WMD: 0.42, (95% CI: 0.03–0.81); *P* = 0.03], duration of gastroenteroscopy [WMD: 0.22; (95% CI: −0.09 to 0.53); *P* = 0.16] and recovery time [WMD: 0.48; (95% CI: 0.13–0.83); *P* = 0.007] were not significantly worse with ciprofol compared to propofol as shown in Figure 3.

**FIGURE 4:**
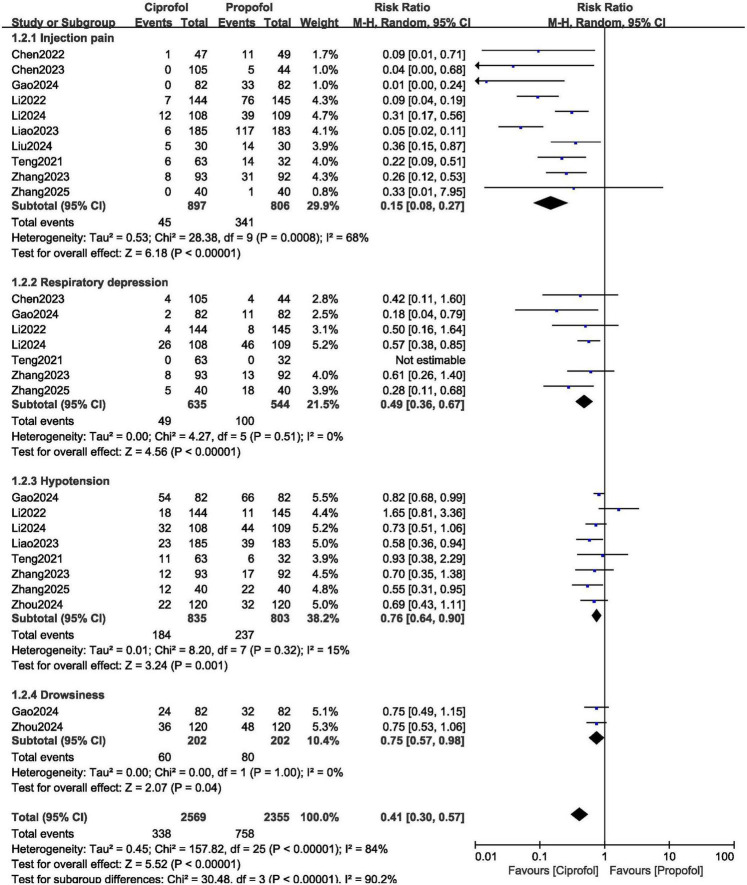
Adverse drug events observed with ciprofol compared to propofol when sedating patients in gastroenteroscopy (Part A).

Ciprofol was associated with a significantly lower risk of injection pain (RR: 0.15, 95% CI: 0.08–0.27; *P* = 0.00001), respiratory depression (RR: 0.49, 95% CI: 0.36–0.74; *P* = 0.0003), hypotension (RR: 0.76, 95% CI: 0.64–0.90; *P* = 0.001) and drowsiness (RR: 0.75, 95% CI: 0.57–0.98; *P* = 0.04) as shown in Figure 4.

**FIGURE 5:**
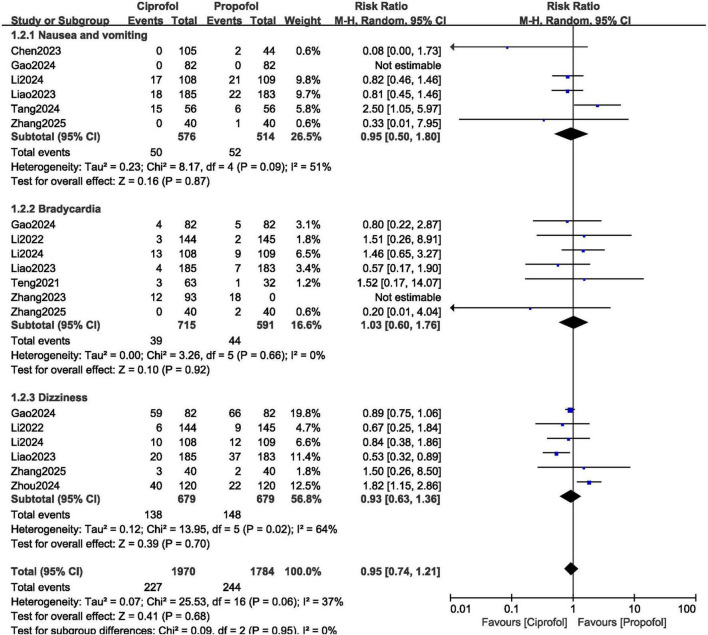
Adverse drug events observed with ciprofol compared to propofol when sedating patients in gastroenteroscopy (Part B).

In addition, the risk for nausea and vomiting (RR: 0.95, 95% CI: 0.50–1.80; *P* = 0.87), bradycardia (RR: 1.03, 95% CI: 0.60–1.76; *P* = 0.92) and dizziness (RR: 0.93, 95% CI: 0.63–1.36; *P* = 0.70) were not increased with ciprofol as shown in Figure 5.

**FIGURE 6:**
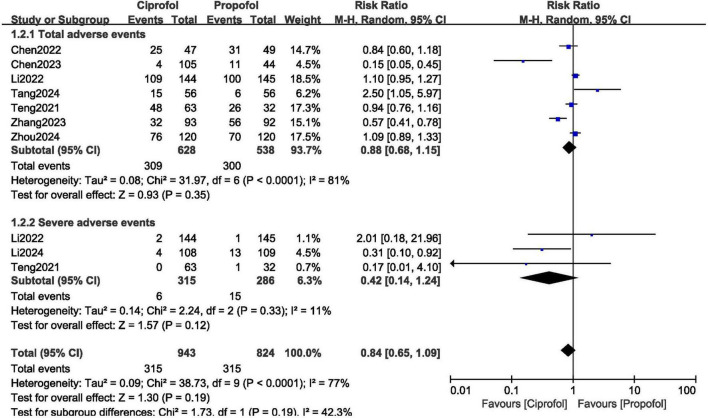
Adverse drug events observed with ciprofol compared to propofol when sedating patients in gastroenteroscopy (Part C).

Total adverse events (RR: 0.88, 95% CI: 0.68–1.15; *P* = 0.35) and serious adverse events (RR: 0.42, 95% CI: 0.14–1.24; *P* = 0.12) were not significantly different with ciprofol versus propofol for sedation in gastroenteroscopy as shown in Figure 6.

Sensitivity analysis showed consistent results throughout. This was carried out by a method of exclusion. One at a time, each original study was excluded and a new analysis was carried out for every subgroup and the new results were compared with the main results of this analysis and any significant change was observed.

The sensitivity analysis was as followed: when study Chen et al. (11) was excluded and a new analysis was carried out, induction time (WMD: 0.10, 95% CI: −0.25 to 0.45; *P* = 0.57), awake time (WMD: 0.22, 95% CI: −0.06 to 0.50; *P* = 0.12), duration of procedure (WMD: 0.10, 95% CI: −0.17 to 0.38; *P* = 0.46) and recovery time (WMD: 0.32, 95% CI: 0.05–0.60; *P* = 0.02) were not significantly different from the main analysis. In addition, when Li et al. (14) was excluded, and a new analysis was carried out, induction time, awake time and operation duration time was still not significantly different. Similar results were obtained when the other studies were excluded.

For the adverse drug events, when study Chen et al. (12) was excluded, injection pain (RR: 0.16, 95% CI: 0.08–0.29; *P* = 0.00001), nausea and vomiting (RR: 1.04, 95% CI: 0.59–1.85; *P* = 0.88), respiratory depression (RR: 0.49, 95% CI: 0.35–0.68; *P* = 0.0001), hypotension (RR: 0.76, 95% CI: 0.64–0.90; *P* = 0.001), and total adverse events (RR: 0.95, 95% CI: 0.77–1.18; *P* = 0.66) were not significantly different from the main results. Similarly, when study Gao et al. (13) was excluded and a new analysis was carried out, the results for injection pain (RR: 0.16, 95% CI: 0.09–0.29; *P* = 0.00001), nausea and vomiting (RR: 0.95, 95% CI: 0.50–1.80; *P* = 0.87), respiratory depression (RR: 0.51, 95% CI: 0.38–0.70; *P* = 0.0001), hypotension (RR: 0.72, 95% CI: 0.58–0.91; *P* = 0.006), bradycardia (RR: 1.08, 95% CI: 0.60–1.96; *P* = 0.79), and dizziness (RR: 0.93, 95% CI: 0.50–1.73; *P* = 0.82) were not significantly different from the main results. Similar consistent results were obtained when the other original studies were excluded by turn and newer analyses were carried out.

**FIGURE 7:**
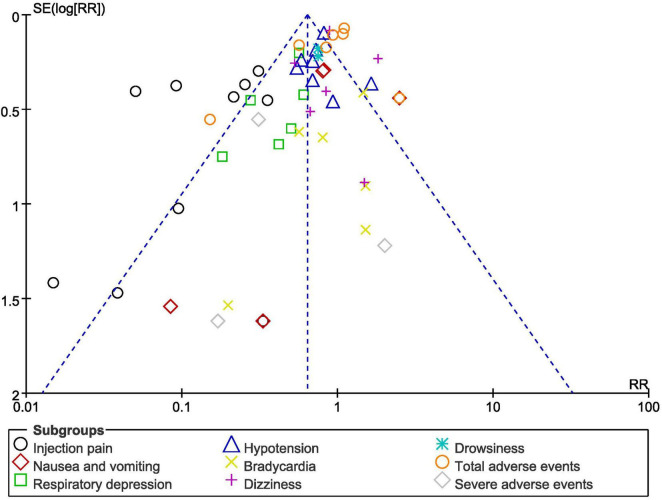
Funnel plot showing publication bias.

Publication bias was visually observed through the Revman generated funnel plot as shown in Figure 7. As shown in Figure 7, there was a low evidence of publication bias across all the original studies that were involved in the comparison of ciprofol versus propofol for gastroenteroscopy.

## Discussion

Propofol, also known as 2,6-diisopropylphenol is considered a potent intravenous hypnotic drug (22). It was developed by the Imperial Chemical Industries Limited in London, and was commercialized in the year 1986 in Europe and 1989 in the United States of America. This intravenous anesthetic agent has a rapid onset, with a smooth induction, and a rapid terminal half-life time and is metabolized almost completely by the liver.

Ciprofol, also known as HSK3486, has been derived from propofol (23). It is a new 2,6 disubstituted phenol derivative which was developed by Haisco Pharmaceutical Group Co, Ltd. in Chengdu, China. Ciprofol was first reported in the year 2017 and was approved by the China National Medical Products Administration (NMPA) for several purposes including endoscopy and general anesthesia. This intravenous drug is extensively metabolized following administration primarily in the liver through phase I cytochrome P450 (CYP) 2B6 and phase II glucuronosyltransferase 1A9 (24).

Through this meta-analysis, we aimed to compare the outcomes observed with ciprofol versus propofol for sedation during gastroenteroscopy in Chinese patients. Our study included above 2,000 participants from Mainland China. The current results showed ciprofol to be at least as effective as propofol during sedation of patients for gastroenteroscopy.

In China, a phase III clinical trial compared the effectiveness of ciprofol with propofol to induce deep sedation for gastroenteroscopy (14). Thirty patients underwent gastroscopy whereas 259 patients underwent colonoscopy. The success rate of gastroscopy was 100% in both groups whereas the success rate of colonoscopy was 100% in the ciprofol group and 99.2% in the propofol group. The mean time for a patient to become fully awake and the time taken to be discharged from the hospital among patients in the ciprofol group were longer than that in the propofol group. However, most of the patients preferred ciprofol in comparison to propofol for gastroenteroscopy. Similarly, in our current analysis, beneficial effects were observed with ciprofol during gastroenteroscopy.

A new randomized, double-blind controlled clinical trial (17) comparing the efficacy and safety of ciprofol, propofol and propofol and etomidate mixture, and ciprofol and etomidate mixture in patients who underwent painless gastroscopy and involving 120 participants, showed ciprofol to have effectively induced sedation during gastroscopy similar to propofol, with comparable safety profile. However, in our current analysis, the induction time was significantly better with propofol when compared to ciprofol.

Moreover, in another study, which was a single-center, placebo-controlled randomized trial (16), including 368 participants who underwent gastroscopy, the authors demonstrated that compared to propofol, ciprofol had significantly lesser impact on hemodynamics, was associated with significantly lower risk of respiratory depression, and less injection pain which would favor its use in painless gastroscopy.

Ciprofol has not only shown to be effective to sedate patients for gastroenteroscopy, but has also been effective in induction and maintenance of general anesthesia during elective surgery (25). A systematic review and meta-analysis compared the efficacy and safety of ciprofol with propofol in sedating patients in the operating room and outside the operating room (26). The authors concluded that the risks of injection site pain and respiratory depression were reduced with ciprofol. In addition, intra-operative hypotension and physical movement were also significantly reduced. However, longer induction and awakening time were observed with ciprofol when compared to propofol.

A protocol for a systematic review and meta-analysis based on ciprofol versus propofol for sedation in gastrointestinal endoscopy was published (27). Ciprofol has high efficacy, good selectivity and fewer associated adverse events indicating good clinical application potential at least in gastroenteroscopy. Future research studies will further focus on this novel anesthetic agent. Furthermore, up to now ciprofol is only approved for use in China. It is not known whether we can count on the study designs and results. Future studies and research carried out in other Non-Chinese countries should be encouraged.

## Limitations

This study also has limitations. First of all, the total number of participants which were used in this analysis was limited around 2,000 which could affect the results of this analysis. However, there were only a few studies with limited number of participants published based on this particular research idea. Another limitation could be the high level of heterogeneity during data analysis. In addition, another limitation was the fact that ciprofol has only been approved in China and this study consisted only of Chinese participants therefore the results might not be generalized and could be limited to only Chinese population. Also, non-English and unpublished data were not included in this analysis and this could be another limitation of this study. Another limitation was the fact that quantitative test like the egger’s test were not used to assess publication bias. Instead, publication bias was only assessed by funnel plots. However, due to the smaller number of studies with small size participant numbers, this egger’s test will not be significant. For smaller number of studies and studies with smaller sample sizes, publication bias could well be assessed through funnel plots. Another limitation could be the fact that factors such as the mean age, the body mass index and the blood pressure varied between the groups, however, they were not adjusted for the analysis. Moreover, lack of subgroup analyses (for example gastroscopy versus colonoscopy) was due to lack of data based on those settings for comparison. Another limitation of this analysis could be variation in drug dosage or procedural protocols among studies. In addition, terms like “awake,” “fully awaking,” “recovery time” could have differently been defined in different studies. This could have lead to shortcomings and affected our results. Hence, it could also be considered a limitation of this analysis.

## Conclusion

Through this meta-analysis, it could be concluded that ciprofol was apparently not associated with significantly worse procedural outcomes nor associated with increased adverse drug events compared to propofol during gastroenteroscopy in Chinese patients. However, in view of several limitations in this analysis, this hypothesis should further be confirmed in future studies.

## Data Availability

The datasets presented in this study can be found in online repositories. The names of the repository/repositories and accession number(s) can be found below: all data are listed in the manuscript. However, references have been provided to the original papers.

## Footnotes

1
http://www.Clinicaltrials.gov
